# Reading skills deficits in people with mental illness: A systematic review and meta-analysis

**DOI:** 10.1192/j.eurpsy.2020.98

**Published:** 2020-11-03

**Authors:** Martina Vanova, Luke Aldridge-Waddon, Ben Jennings, Ignazio Puzzo, Veena Kumari

**Affiliations:** 1 Centre for Cognitive Neuroscience, College of Health, Medicine and Life Sciences, Brunel University London, Uxbridge, United Kingdom; 2 Division of Psychology, Department of Life Sciences, College of Health, Medicine and Life Sciences, Brunel University London, Uxbridge, United Kingdom

**Keywords:** Cognition, forensic psychiatry, personality disorders, reading skills, schizophrenia

## Abstract

**Background:**

Good reading skills are important for appropriate functioning in everyday life, scholastic performance, and acquiring a higher socioeconomic status. We conducted the first systematic review and meta-analysis to quantify possible deficits in specific reading skills in people with a variety of mental illnesses, including personality disorders (PDs).

**Methods:**

We performed a systematic search of multiple databases from inception until February 2020 and conducted random-effects meta-analyses.

**Results:**

The search yielded 34 studies with standardized assessments of reading skills in people with one or more mental illnesses. Of these, 19 studies provided data for the meta-analysis. Most studies (*k* = 27; meta-analysis, *k* = 17) were in people with schizophrenia and revealed large deficits in phonological processing (Hedge’s *g* = −0.88, *p* < 0.00001), comprehension (Hedge’s *g* = −0.96, *p* < 0.00001) and reading rate (Hedge’s *g* = −1.22, *p* = 0.002), relative to healthy controls; the single-word reading was less affected (Hedge’s *g* = −0.70, *p* < 0.00001). A few studies in affective disorders and nonforensic PDs suggested weaker deficits (for all, Hedge’s *g* < −0.60). In forensic populations with PDs, there was evidence of marked phonological processing (Hedge’s *g* = −0.85, *p* < 0.0001) and comprehension deficits (Hedge’s *g* = −0.95, *p* = 0.0003).

**Conclusions:**

People with schizophrenia, and possibly forensic PD populations, demonstrate a range of reading skills deficits. Future studies are needed to establish how these deficits directly compare to those seen in developmental or acquired dyslexia and to explore the potential of dyslexia interventions to improve reading skills in these populations.

## Introduction

Reading is a complex process that requires the implementation of various skills simultaneously. To begin with, it requires recognition of the visual information necessary to extract the information from text [1]. The core reading skill is phonological processing, which involves recognition of the sound structure of the language, the decoding of written symbols into sounds (phonological awareness), and then their maintenance in working memory (phonological memory) [2]. Phonological processing facilitates the decoding of written information, which leads to word identification and subsequent extraction of meaning [3]. A failure to read each word correctly leads to problems with comprehension [4] as comprehension involves the processing of individual letters and words, and then putting them together to form meaning [5]. When one or more of these reading skills are impaired, and this impairment cannot be explained by general cognitive dysfunction or intelligence, this is referred to as dyslexia [6]. Overlaps between dyslexia and schizophrenia (SZ) have been suggested, based on previous findings of disruption in the processes that support skilled reading (e.g., deficits in language, auditory and visual perception, oculomotor control) in both disorders [7] but the nature and severity of reading skills deficits in SZ and other severe mental illnesses (MIs) remain unclear at present.

Reading skills are of enormous significance for a range of socioeconomic outcomes in modern societies, including academic performance, occupational achievement, and family and social relationships [8,9]. Furthermore, poor reading skills in children have been associated with increased antisocial behavior [10,11]. Likewise, in forensic populations, poor reading skills and dyslexia traits have been associated with increased anxiety and poor socialization, which, in turn, might explain their antisocial behavior [12,13]. In people with various MIs, undiagnosed reading problems, and dyslexia result in scholastic failure, in turn raising the risk for mood problems [14] and future criminal behavior [15]. Poor reading skills also pose a challenge for accessibility of mental health interventions [16] and predict poor psychosocial outcomes [17,18]. There is thus a need to consider reading deficits as a therapeutic target and address them, for example, with interventions used for dyslexia [7,19]. A thorough understanding of the pattern and magnitude of reading deficits in people with specific MIs is an important first step toward this goal.

The main aim of this systematic and meta-analytic review was to conduct a comprehensive analysis to delineate the nature and magnitude of reading impairments based on data from studies that employed standardized tools to assess reading skills in people with SZ, bipolar disorder, affective disorders (major depression, anxiety, mania), personality disorders (PDs; borderline personality disorder [BPD], antisocial personality disorder [ASPD], psychopathy), and general MIs (across diagnoses/not-specified). Our secondary aims were to examine whether (a) particular reading skill deficits were more strongly present when assessed with some tests compared to others, given that reading skills in different studies have been quantified using a variety of tests and batteries, and (b) groups with MIs and a forensic history show more pronounced deficits relative to those from nonforensic settings.

## Methods

This systematic literature review and meta-analysis followed PRISMA guidelines [20]. Search terms and key articles were identified based on an exploratory search of databases and an internet search engine (Google Scholar). We then searched Academic Search Complete, CINAHL Plus, PsycINFO, PsycARTICLES, SocINDEX, MEDLINE via EBSCO Host and PubMed (up to Feb 2020) for all studies including reading assessment(s) in MIs (see Table 1 for the full search strategy and eligibility criteria). Manual searches were conducted using the relevant literature [7,17,21].

**Table 1.** Full search strategy per database and eligibility criteria.

EBSCO search: *Academic Search Complete, CINAHL Plus, PsycINFO, PsycARTICLES, SocINDEX, MEDLINE*	PubMed
(Reading* OR literacy OR scholastic) AND (schizophren* OR “schizoaffective disorder” OR psychosis OR psychotic OR bipolar OR psychopathy OR “personality disorder” OR “antisocial personality disorder” OR “mental disorder” OR “mental ill*” OR “mood disorder” OR “anxiety” OR depress*) AND adult* (Dyslexia OR “learning disability” OR “reading disorder” OR “reading dysfunction” OR “reading deficit”) AND (schizophren* OR “schizoaffective disorder” OR psychosis OR psychotic OR bipolar OR psychopathy OR “personality disorder” OR “antisocial personality disorder” OR “mental disorder” OR “mental ill*” OR “mood disorder” OR “anxiety” OR depress*) AND adult* *Related words and related subjects, only peer-reviewed.	(((((((“Mental Disorders”[Mesh]) OR (“Schizophrenia”[Mesh] OR “Schizophrenia Spectrum and Other Psychotic Disorders”[Mesh] OR “Schizophrenia, Paranoid”[Mesh] OR “Schizophrenia, Disorganized”[Mesh] OR “Schizophrenia, Catatonic”[Mesh] OR “Schizotypal Personality Disorder”[Mesh])) OR “Psychotic Disorders”[Mesh]) OR (“Bipolar Disorder”[Mesh] OR “Depressive Disorder, Major”[Mesh] OR “Major Affective Disorder 1” [Supplementary Concept] OR “Major Affective Disorder 2” [Supplementary Concept])) OR “Antisocial Personality Disorder”[Mesh]) OR (“Personality Disorders”[Mesh] OR “Schizoid Personality Disorder”[Mesh] OR “Passive-Aggressive Personality Disorder”[Mesh] OR “Paranoid Personality Disorder”[Mesh] OR “Multiple Personality Disorder”[Mesh] OR “Histrionic Personality Disorder”[Mesh] OR “Dependent Personality Disorder”[Mesh] OR “Compulsive Personality Disorder”[Mesh] OR “Borderline Personality Disorder”[Mesh])) AND (“Reading”[Mesh] OR “Dyslexia”[Mesh] OR “Dyslexia, Acquired”[Mesh])) AND (“Adult”[Mesh] OR “Young Adult”[Mesh]) (((“mood disorders”[MeSH Terms] OR (“mood”[All Fields] AND “disorders”[All Fields]) OR “mood disorders”[All Fields] OR (“mood”[All Fields] AND “disorder”[All Fields]) OR “mood disorder”[All Fields]) OR (“anxiety”[MeSH Terms] OR “anxiety”[All Fields])) AND (“Reading”[Mesh] OR “Dyslexia”[Mesh] OR “Dyslexia, Acquired”[Mesh])) AND (“Adult”[Mesh] OR “Young Adult”[Mesh])
Inclusion criteria	
Case–control, cohort, and cross-sectional studies reporting measures assessing reading abilities in adults with psychosis, depression, anxiety, personality disorders, antisocial personality disorder, psychopathy, and/or general mental illness. Studies using standardized tests and/or translated versions of these into their national language. Quantitative studies published in peer-reviewed journals in English, without publication date restrictions. Abstract and full-text available.	
Exclusion criteria	
Nonpeer reviewed articles, case studies, theses, books, editorial letters, descriptive articles, conference papers, personal opinions, and protocols were excluded. Studies using experimental methods to assess reading in people with MI without reporting scores from standardized tests or Single-word reading tests only to assess premorbid IQ were excluded.	

Abbreviations: MI, Mental Illness; Intelligence Quotient, IQ

Two independent reviewers selected the studies (MV, BJ), and extracted and reviewed data for inconsistencies to reach a consensus (MV, LAW). Extracted data included tests and measures (Table 2), as well as participant characteristics, main findings, the language of assessment, and country (Table 3).

**Table 2.** Tests and measures used in the selected studies (*k* = 34) and diagnoses assessed. Studies involving forensic populations are in *italics*.

Measures (test - subtest name)	Measure description	Used by	Diagnoses assessed
*Phonological processing and decoding*			
Auditory blending test [22]	*Pronounce sounds separately and put them together to form a word.*	Walder et al. [22]	SZ
CTOPP-PA [23]	*Manipulate with sounds, distinguish, pronounce, and synthesize sounds to create words.*	Arnott et al. [24]; Revheim et al. [21]; Revheim et al. [17]; Whitford et al. [7]; Dondé et al. [18]	SZ, SZAD
CTOPP-PM [23]	*Remember and reproduce digits and pronounce nonwords.*	Arnott et al. [24]; Revheim et al. [21]; Revheim et al. [17]; Whitford et al. [7]; Dondé et al. [18]	SZ, SZAD
CTOPP-RN [23]	*Name objects and colours as quickly as possible.*	Arnott et al. [24]; Revheim et al. [21]; Revheim et al. [17]; Whitford et al. [7]	SZ, SZAD
CTOPP-APA [23]	*Manipulate with sounds, distinguish, pronounce, and synthesize sounds to create nonwords.*	Arnott et al. [24]; Revheim et al. [21]; Revheim et al. [17]; Dondé et al. [18]	SZ, SZAD
CTOPP-ARN [23]	*Name letters and numbers as quickly as possible.*	Revheim et al. [21]; Revheim et al. [17]	SZ, SZAD
JDT [25] (Wordchains)	*Decode words from a group of letters and mark a space between them (e.g., girl/chair/meet).*	*Daderman et al.* [15]*; Selenius et al.* [26]*; Svensson et al.* [27]	PD, MI
MWDT [28]	*Read specific words.*	*Selenius et al.* [26]	MI
PALPA [29]	*Nonword judgments or segment words/nonwords.*	*Brites et al.* [30]*; Selenius et al.* [26]	Psychopathy, MI
Phonological choice [31]	*Decide which nonword in a pair sounds like a real word.*	*Svensson et al.* [27]	MI
RAN [32]	*Name the letters, numbers, colours, or pictures presented on cards.*	Walder et al. [22]	SZ
RNRT, RNST [33]	*Read or spell a list of nonwords and identify words read to the subject each syllable separately.*	Walder et al. [22]	SZ
The Pidgeon [34]	*Five tasks: self-reported dyslexic problems, working memory, vocabulary, reversed spoonerism, phonological choice, and orthographic choice.*	*Selenius et al.* [26]	MI
WJTA-III [35]	*Read or spell a list of nonwords.*	Leonard et al. [36]; Revheim et al. [21]; Revheim et al. [17]	SZ, SZAD
WRMT-R [37] (Word attack)	*Read as many nonwords as possible in 1 min.*	*Svensson et al.* [27]	MI
*Comprehension*			
BDAE [38]	*Answer questions (multiple-choice) about a text.*	Gavilán and García-Albea [39]	SZ
GORT-4 [40]	*Respond to questions about the block of text read.*	Martinez et al. [41]; Revheim et al. [21]; Revheim et al. [17]	SZ, SZAD
ITBS [42], ITED [43]	*Comprehension of fiction and nonfiction text.*	Fuller et al. [44]; Ho et al. [45]	SZ
Israeli language skills test [46]	*Comprehension of ideas presented in a block of text of increasing difficulty.*	Reichenberg et al. [47]	SZ, SZAD, BD
NARA-III [48]	*Respond to open questions about the block of text read.*	Arnott et al. [24]	SZ
NDRT [49]	*Respond to questions about the block of text read.*	Revheim et al. [21]; Revheim et al. [17]; Whitford et al. [7]	SZ, SZAD
PIAT [50]	*Use pictures to describe the meaning of a sentence.*	Berg and Hammitt [51]	MI
PALPA	*Choose a picture which fits the meaning of a sentence or a word.*	*Brites et al.* [30]; Gavilán and García-Albea [39]	Psychopathy, SZ
RAN	*Reproduce letters and digits.*	*Svensson et al.* [27]	MI
RCBA [52], RCBA-2 [53]	*10 subscales (I-X). Answer questions (multiple-choice, silent reading) about single words, sentences, paragraphs, functional information, synonyms.*	Arnott et al. [24]; Hayes and O’Grady [54]	SZ
“Summer with Monika” [55]	*“Fill in the blank” response about a text.*	*Daderman et al.* [15]	PD
“The Hedgehog” [56]	*Underline a salient word in a text.*	*Selenius et al.* [26]	MI
WJTA-III	*“Fill in the blank” response about a text.*	Leonard et al. [36]; Revheim et al. [21]; Revheim et al. [17]; Dondé et al. [18]	SZ, SZAD
WRAT-IV [57]	*Complete a sentence with an appropriate word.*	Ferron et al. [58]; Patrick et al. [59]	MI, SZ
WRMT-R	*Text passages followed by a blank line to orally fill in a word that fits the passage.*	Arnott et al. [24]; *Svensson et al.* [27]	SZ, MI
Paragraph reading [60]	*Answer questions (Yes/No, and multiple choice) about a block of text.*	Disimoni et al. [60]	SZ
*Single-word reading*			
LNNB [61]	*A comprehensive battery assesses various neuropsychological functions, including reading.*	Maj [62]; Puente et al. [63]	SZ, SZAD, DD
MWDT	*Read specific words out loud.*	*Daderman et al.* [15]	PD
PALPA	*Read letters, syllables, words, and sentences out loud.*	*Brites et al.* [30]	Psychopathy
PIAT	*Read individual words out loud.*	Berg and Hammitt [51]	MI
REALM [64]	*Pronounce words commonly used in medicine. A scale from 3rd grade and up to high school reading performance.*	Christensen and Grace [65]; Weiss et al. [66]	MI, DD
TOWRE [67]	*Read individual words out loud.*	*Davidson et al.* [68]	ASPD
WJTA-III	*Read individual words out loud.*	Leonard et al. [36]; Dondé et al. [18]	SZ, SZAD
WRAT [69]	*Read individual words out loud.*	*Dalby and Williams* [70]*; Nestor* [71]; Revheim et al. [21]; Walder et al. [22]; Nelson et al. [72]; Potter and Nestor [73]; Ferron et al. [58]; Light et al. [74]; Martínez et al. [41]; Revheim et al. [17]	SZ, SZAD, MI
WRMT-R	*Read individual words/nonwords out loud.*	Arnott et al. [24]	SZ
*Rate*			
Alouette [75]	*Total number of words correctly read.*	Curzietti et al. [76]	SZ
GORT-4	*Time taken to read a block of text.*	Revheim et al. [21]; Revheim et al. [17]	SZ, SZAD
NARA-III	*Number of words read per minute.*	Arnott et al. [24]	SZ
NDRT	*Number of words read in the first min.*	Whitford et al. [7]	SZ
*Speed*			
Alouette	*Overall reading time (max. 180 s.).*	Curzietti et al. [76]	SZ
“Summer with Monika”	*Overall reading time of the text.*	*Daderman et al.* [15]	PD
“The Hedgehog”	*Overall reading time of the text.*	*Selenius et al.* [26]	MI
RCBA-2	*Overall completion time of 10 tasks.*	Arnott et al. [24]	SZ
*Accuracy*			
Alouette	*Number of words correctly read in 180 s. limit.*	Curzietti et al. [76]	SZ
GORT-4	*Number of correctly/incorrectly read words.*	Revheim et al. [21]; Revheim et al. [17]	SZ, SZAD
NARA-III	*Number of errors made when reading a block of text.*	Arnott et al. [24]	SZ
*Fluency*			
GORT-4	*Sum of rate and accuracy scores.*	Revheim et al. [21]; Martinez et al. [41]; Revheim et al. [17]	SZ, SZAD
“Arthur the Young Rat” [77]	*Number of nonfluencies in text reading (i.e., repetitions of a sound, syllable, word, or phrase).*	Halpern et al. [77]	SZ
“Grandfather” [77]		Halpern et al. [77]	SZ
BDAE		Halpern et al. [77]	SZ
Fisher-Logemann [78]		Halpern et al. [77]	SZ
WJTA-III	*Time taken to read a block of text followed by questions.*	Revheim et al. [17]; Dondé et al. [18]	SZ, SZAD
*Vocabulary*			
ITBS, ITED	*Select a word or phrase synonymous to the target word.*	Fuller et al. [44]; Ho et al. [45]	SZ
NDRT	*Answer multiple-choice questions about words.*	Revheim et al. [21]; Revheim et al. [17]	SZ, SZAD
MSVT [56]	*Find word’s synonym among five options.*	*Selenius et al.* [26]	MI
*Spelling*			
ITBS, ITED	*Spelling of real word by writing.*	Fuller et al. [44]	SZ
MST [56]	*Spelling of real word by writing.*	*Daderman et al.* [15]*; Selenius et al.* [26]	PD, MI
Orthographic choice [31]	*Decide which of the two words presented is correctly spelt.*	*Svensson et al.* [27]	MI
WJTA-III	*Spelling of real words out loud or by writing.*	Revheim et al. [17]	SZ, SZAD
*Grammar*			
Caplan and Hildebrandt task [79]	*Identify the subject and object of the actions of phrases.*	Walder et al. [22]	SZ
*Orthography*			
Pseudo-homophone discrimination [80], Animal word cross-out test [80], onset judgment test [80]	*Mark particular words/nonwords within a time limit.*	Wang et al. [81]	SZ

Abbreviations: BD, Bipolar Disorder; BDAE, Boston Diagnostic Aphasia Examination; CTOPP, Comprehensive Test of Phonological Processing (PA, Phonological Awareness; PM, Phonological Memory; RN, Rapid Naming; APA, Alternative Phonological Awareness; ARN, Alternative Rapid Naming); DD, Depressive Disorder; GORT, Gray Oral Reading Test; HC, Healthy Controls; ITBS, Iowa Test of Basic Skills; ITED, Iowa Test of Educational Development; JDT, Jacobson’s Decoding Test; LNNB, Luria-Nebraska Neuropsychological Battery; MI, Mental Illness; MST, Madison’s Spelling Test; MSVT, Madison’s Standardized Vocabulary Test; MWDT, Madison’s Word Decoding Test; NARA, Neale Analysis of Reading Ability; NDRT, Nelson–Denny Reading Test; PALPA, Psycholinguistic Assessments of Language Processing in Aphasia; PD, Personality Disorder; PIAT, Peabody Individual Achievement Test; RAN, Rapid Automatised Naming; RCBA, Reading Comprehension Battery for Aphasia; REALM, Rapid Estimate of Adult Literacy in Medicine; RNRT, Roentgen’s Nonwords Reading Test; RNST, Roeltgen’s Nonwords Spelling Test; SZ, Schizophrenia; SZAD, Schizoaffective Disorder; TOWRE, Test of Word Reading Efficiency; WJTA-III, Woodcock–Johnson III Tests of Achievement (BR, Broad Reading; BRS, Basic Reading Skills; RC, Reading Comprehension; PKG, Phoneme-Grapheme Knowledge); WRAT, Wide Range Achievement Test; WRMT-R, Woodcock Reading Mastery Test—Revised (BS, Basic Skills; PC, Passage Comprehension; PKG, Phoneme-Grapheme Knowledge).

**Table 3:** Summary of key data extracted from selected studies (*k* = 34).

1. Psychosis											
Study	Dg.	Sample (N) (M/F)	Age (Mean, SD)	Medication (mg/day, CPZE)	Education years (Mean, SD)	Tests (subtests)	Variables examined	Reading performance	Symptoms, medication and reading	Cognition, education and reading	Language
Disimoni et al. [60]	**SZ**	SZ = 27 (9/18)	SZ = 36.3 (13.2)	NR	SZ = 11.3 (2.6)	Language battery: comprehension (3 subtests), naming, writing, arithmetic	comprehension	SZ was impaired in comprehension but less than aphasics. Poorer speaking and listening scores were linked with better reading. This indicated independence of communication skills from reading.	NE	NE	English
Maj [62] *Meta-analysis*	**SZ, SZAD, DD**	SZAD = 16 (7/9); SZ = 20 (8/12); DD = 16 (7/9); ex SZAD = 15 (7/8); **HC = 20** (8/12)	SZAD = 33.6 (6.1), DD = 36.5 (6.9), SZ = 31.7 (8.9), HC = 33.5 (5.8), exSzAD = 36.5 (5.6), HC = 37.7 (5.9)	Lithium <1200, antidepressants <75, and/or haloperidol <5 or chlorpromazine <100	NR	LNNB (reading: 13 items)	single-word reading	SZ scored significantly worse than HC in reading. SZ also demonstrated (nonsignificantly) worse reading skills than the SZAD and the DD.	NE	Means for cognitive domains were reported but the relationship with reading NE. Groups did not differ in years of education.	Italian
Halpern et al. [77]	**SZ**	SZ = 7 (7/0); Atypical Organic Brain Syndrome =1 (1/0)	SZ = 51.5	NR	NR	BDAE (subtest L), Fisher-Logemann sentences, “Grandfather” passage, “Arthur the Young Rat” passage	reading fluency of words, sentences, and paragraphs	No significant amount of nonfluencies in reading were found based on location in a sentence, location in the utterance (sound, syllable, word, phrase, and sentence) or symptoms (repetitions, prolongations, hesitations). Significantly more nonfluencies occurred in sentence reading and paragraph reading and in the middle and beginning of sentences.	NE	NE	English
Puente et al. [63]	**SZ**	SZ total = 60; SZ-brain damage = 20 (15/5); nonbrain damage = 20 (15/5); acute = 20 (11/9); **HC = 20** (6/14)	SZ-brain damage = 51.7 (17.8) nonbrain damage = 36.1 (11.1) acute = 34.5 (14.2) **HC = 19.5** (2.1)	SZ-brain damage = 405.0; CPZE nonbrain damage = 234.8; CPZE acute = 492.2; CPZE	SZ-brain damaged = 9.8 (2.6); Nonbrain damaged = 10.7 (2.4); acute = 11.4 (3.1); HC = 12.6 (1.1)	LNNB	single-word reading	No significant differences between SZ and HC.	No significant correlation between medication dosage and LNNB battery. Other relationships NE.	NE	English
Fuller et al. [44]	**SZ**	SZ = 70 (57/13)	SZ = 28.0 (6.9)	NR	NR	ITBS, ITED	comprehension, spelling, language, vocabulary	SZ scores were significantly lower than average general rank between 11th grade and the 4th and 8th grade respectively in reading, vocabulary, language, and other scholastic skills. Reading performance significantly dropped between grades 8 and 11. ITED scores did not predict the age of onset of SZ.	NE	WAIS-R verbal IQ significantly positively correlated with reading, vocabulary and language skills measured by ITED in 11th grade in SZ.	English
Reichenberg et al. [47]	**SZ, SZAD, BD**	SZ = 536 (390/146); SZAD = 31 (23/8); BD = 68 (38/30); **HC = 635** (451/184)	SZ = 20.7 (2.0); SZAD = 20.0 (1.5); BD = 21.5 (2.8)	NR	NR	Israeli language skills assessment (2 subtests)	comprehension, reading sentences	SZ but not BD had significantly worse scores in reading and reading comprehension in comparison with HC.	NE	NE	Israeli
Hayes and O’Grady [54] *Meta-analysis*	**SZ**	SZ = 30 (26/4); **HC = 30** (26/4)	SZ = 37.3 (11.20); HC = 37.2 (11.85)	NR	NR	RCBA (10 subtests)	single-word comprehension, functional reading, comprehension of synonyms, sentence comprehension, paragraph comprehension, factual comprehension, inferential reading, comprehension with structure variation, reading speed	SZ scored lower in comprehension (9/10 RCBA subtests were significantly lower in SZ) than HC but retained word-recognition skills (NART). Reading time is longer in SZ. Functional reading necessary for real-life functioning was significantly impaired in SZ.	NE	Lower premorbid IQ (NART) correlated with low RCBA scores. Education levels for each group were similar.	English
Ho et al. [45] *Meta-analysis*	**SZ**	SZ = 70 (57/13); comparison subjects =147 (**HC = 36:** Alc = 66.7% drug = 34.7% DD = 29.9%) (63/84)	NR	NR	NR	ITBS, ITED	comprehension, vocabulary	SZ patients scored lower in all subtests than comparisons. However, tests had poor screening efficiency for SZ due to low positive predictive values. Reading in SZ was lower than in comparison group in all grades (4th, 8th, and 11th), lowest in 11th grade. Effect sizes were reduced when gender and parental social-economic status were accounted for.	NE	NE	English
Revheim et al. [21] *Meta-analysis*	**SZ, SZAD**	SZ/SZAD = 19 (18/1); **HC = 10** (6/4)	SZ = 38.3 (9.6); HC = 28.7 (9.0)	1077.7 ± 574 CPZE	SZ = 12.4 (2.3); HC = 15.2 (0.85)	GORT-4, CTOPP (12 subtests), WJTA-III (7 subtests), NDRT (3 subtests), WRAT-III	GORT: comprehension, rate, accuracy, fluency, ORQ; CTOPP: PA, PM, RN, APA, ARN WJTA-III: (BR) - reading decoding, speed, comprehension/(BRS) - vocabulary, phonics, structure/(RC) - comprehension, vocabulary, reasoning/(PGK) - phonic and orthographic processes; NDRT: vocabulary, comprehension and total score; WRAT-III: single-word reading	SZ show significantly impaired reading abilities than HC. Patients’ reading levels were 3.4 years below their education level. Significant differences between SZ and HC were in all subtests except in CTOPP-RN and NDRT-PKG. No differences between SZ and HC in WRAT scores.	PANSS-Cog negatively correlated with GORT-4 comprehension. Relationship between medication and reading NE.	WAIS-III working memory or processing speed could not predict GORT-4 scores. Groups differed significantly in education. Sz had reading 3.4 years below achieved education years.	English
Walder et al. [22] *Meta-analysis*	**SZ**	SZ = 31 (17/14); **HC = 27** (13/14)	SZ = 39.1 (7.0)	520 ± 428 CPZE	NR	RNRT, RNST, Auditory blending test, RAN, Caplan and Hildebrandt’s task, WRAT-R	RNRT, RNST, auditory blending test & RAN: all phonological processing; WRAT-R: single-word reading; Caplan and Hildebrandt’s task: grammar	Women with SZ had relatively preserved phonology and grammar function when compared with HC women. SZ men generally impaired in language skills in comparison with HC men, especially in phonology and grammar. Men and women with SZ differed most in grammar. Sex and group had a significant effect on phonology and grammar.	No significant differences in chlorpromazine levels. Relationship between symptoms and reading NE.	Attention scores entered as a covariate in the analysis. Relationship between education and reading NE.	English
Nelson et al. [72]	**SZ**	SZ = 100 (72/28)	SZ = 38.28 (9.37)	795.80 ± 566.16 CPZE	SZ = 12.31 (9.10)	WRAT-III	single-word reading	SZ scored *M* = 78.00 (21.01) in WRAT. Relationship between premorbid functioning (WRAT) and social cognition is unclear.	No significant correlation between BPRS scores and WRAT. Relationship between medication and reading NE.	NE	English
Leonard et al. [36] *Meta-analysis*	**SZ**	SZ = 45 (36/9); **HC = 39** (36/3)	SZ = 41.1 (10); HC = 42.0 (10)	NR	NR	WJTA-III (3 subtests)	Word attack: phonological decoding; Letter-Word Identification: single-word reading (word recognition); Passage comprehension: comprehension	SZ scored significantly lower than HC in phonological decoding, comprehension, and single-word reading. Anatomical risk index predicted 38% of the variance in verbal ability and 44% of the variance in comprehension.	NE	Broad cognitive ability was significantly lower in SZ, but no correlations with reading skills were reported. Relationship between education and reading NE.	English
Potter and Nestor [73] *Meta-analysis*	**SZ**	SZ-Preserved =21 (19/2); SZ-Deteriorated = 21 (16/5); SZ-Compromised = 31 (23/8); **HC = 74** (47/27)	SZ-P = 36.31 (11.06); SZ-D = 41.40 (10.42); SZ-C = 38.71 (10.93); HC = 40.59 (8.89)	410.70 ± 298.76 CPZE	SZ-P = 13.7 (1.809); SZ-D = 13.214 (1.29); SZ-C = 12.18 (1.98); HC = 15.27 (2.029)	WRAT-III	single-word reading	SZ-compromised scored significantly lower than all other groups. No significant differences between other SZ groups and HC.	NE	Significant differences were found between the SZ IQ subgroups in memory and executive functioning. No correlation with reading was reported. Relationship between education and reading NE.	English
Arnott et al. [24] *Meta-analysis*	**SZ**	SZ = 16 (10/6); **HC = 12** (6/6)	SZ = 41.19 (13.43); HC = 42.17 (15.56)	417.86 ± 375.22 CPZE	SZ = 11.88 (1.78); HC = 11.75 (2.18)	NARA-III; WRMT-R (3 subtests), RCBA-2 (10 subtests), CTOPP (8 subtests)	NARA-III: comprehension, rate, accuracy; WRMT-R: comprehension, word recognition (Basic Skills subscore); RCBA-2: comprehension, total time; CTOPP: PA, APA, PM, RN	SZ had impaired comprehension and rate in NARA. Phonological processes were related to symptomatology but only CTOPP-RN was significantly lower in SZ than HC. Reading comprehension measured by RCBA was mostly spared in SZ. Reading words and nonwords was comparable in SZ and HC.	PANSS-N and PANSS-G negatively correlated with CTOPP RN. PANSS-P negatively correlated with CTOPP-PA. Relationship between medication and reading NE.	No significant differences between groups in education. Relationship between cognition and reading NE.	English
Gavilán and Garcia-Albea [39] *Meta-analysis*	**SZ**	SZ = 22 (18/4); **HC = 22** (18/4)	SZ = 42.82 (10.84); HC = 41.95 (10.78)	833.46 CPZE	SZ = 10.18 (2.38); HC = 10.05 (2.44)	PALPA-computerized (comprehension of words and sentences), BDAE (paragraph comprehension), experimental test of figurative language comprehension.	PALPA, BDAE: reading comprehension (words, sentences, paragraphs); experimental: comprehension of metaphors, ironies, proverbs	SZ patients had difficulties in understanding the theory of Mind, which was closely related to the understanding of figurative language. SZ understood proverbs (in isolation) less than ironies and less than metaphors (in context). All figurative language significantly impaired in SZ when compared to HC.	NE	Groups significantly differed in IQ but not premorbid IQ. IQ was a covariate in the analysis. Relationship between education and reading NE.	Spanish
Light et al. [74] *Meta-analysis*	**SZ**	SZ = 341; **HC = 205** (all: 247/94)	SZ = 45.49 (9.37)	NR	SZ = 11.98 (1.99)	WRAT-III	single-word reading	SZ scored significantly lower in WRAT reading than HC at baseline and after 1 year.	NE	NE	English
Martinez et al. [41] *Meta-analysis*	**SZ, SZAD**	SZ = 21; SZAD = 5 (20/5); **HC = 17** (15/2)	SZ/SZAD = 39.4 (10.8); HC = 32.7 (11.0)	1314.1 ± 973.5 CPZE	SZ = 12.4 (2.3); HC = 16.1 (2.4)	GORT-4, WRAT-III	GORT-4: comprehension, fluency (rate + accuracy), ORQ; WRAT-III: single-word reading	SZ scored significantly lower than HC in all passage reading measures. These impairments correlated with reduced fMRI activation in low spatial frequency (LSF) regions (dorsal stream visual system). Deficits in comprehension were greater than in single-word reading.	Reading negatively correlated with antipsychotic dosage. Relationship between symptoms and reading NE.	General intelligence did not predict reading scores. Group differences in reading ability remained when cognitive deficits (processing speed and working memory) were accounted for analyses. Reading was at the 6th-grade level despite achieved 12.4 years of education.	English
Whitford et al. [7] *Meta-analysis*	**SZ**	SZ = 20 (16/4); **HC = 16** (13/3)	SZ = 31.05 (9.08); HC = 31.56 (10.08)	443.57 ± 277.55 CPZE	SZ = 11.85 (1.99); HC = 13.66 (1.87)	CTOPP (6 subtests), NDRT (2 subtests)	CTOPP: PA, PM, RN; NDRT: comprehension, rate	SZ scored significantly lower than HC in all reading measures.	No influence of medication on reading. Relationship between symptoms and reading NE.	Education in years entered as a covariate.	English
Revheim et al. [17] *Meta-analysis*	**SZ, SZAD**	SZ = 37; SZAD = 8 (40/5); **HC = 24** (17/7)	SZ/SZAD = 37.6 (11.6); HC = 39.6 (11.3)	944.3 ± 702.7 CPZE	SZ/SZAD = 12.7 (2.2); HC = 14.6 (1.8)	GORT-4, CTOPP (12 subtests), WJTA-III (7 subtests), NDRT (2 subtests) WRAT	GORT: rate, accuracy, fluency, comprehension; CTOPP: PA, APA, RN, ARN; WJTA-III: fluency, spelling, (BR) - reading decoding, speed, comprehension/(BRS) - vocabulary, phonics, structure/(RC) - comprehension, vocabulary, reasoning/(PGK) - phonic and orthographic processes; NDRT: comprehension, vocabulary, total score; WRAT: single-word reading	Reading skills (GORT-4, CTOPP - APA, RN, ARN, and WJTA-III) were significantly reduced in all SZ in comparison with HC, and significantly below than would be expected based on their general cognition. 73% of SZ met the criteria for dyslexia. WRAT scores were relatively intact in SZ.	No correlation between PANSS scores and reading. Reading deficits positively correlated with the gap between their and parental socioeconomic status. No correlation between medication and reading.	Passage reading was significantly reduced relative to premorbid IQ measured by WRAT. Reading was significantly below achieved education level.	English
Patrick et al. [59] *Meta-analysis*	**SZ**	SZ = 29 (26/3); **HC = 29** (15/14)	SZ = 44.77 (8.24); HC = 40.93 (9.02)	NR	SZ = 13.33 (1.75); HC = 15.34 (2.32)	WRAT-IV	comprehension	SZ patients scored significantly lower in comprehension than HC.	NE	NE	English
Wang et al. [81]	**SZ**	SZ = 22 (12/10); **HC = 22** (13/9)	SZ = 24.36 (4.03); HC = 23.14 (1.94)	582.16 CPZE	SZ = 14.77 (1.06); HC = 15.00 (0.01)	Nonword cross-out test, onset judgment test, animal word cross-out test, pseudo-homophone discrimination test	Nonword cross-out test: orthography onset judgment test: orthography-phonology animal word cross-out test: orthography-semantics (comprehension) pseudo-homophone discrimination test: vocabulary	SZ had impaired all orthographic skills in Chinese while their access to mental lexicon was intact. Reading in Chinese requires also deep orthographic processing which results in impaired reading in Chinese in SZ and this correlated with the severity of psychosis symptoms.	BPRS scores negatively correlated with orthography and orthography-semantics. Relationship between medication and reading NE.	Groups did not differ in achieved education levels. Relationship between cognition and reading NE.	Chinese
Curzietti et al. [76] *Meta-analysis*	**SZ**	SZ = 22 (13/9); **HC = 22** (13/9)	SZ = 41.0 (8.84); HC = 40.03 (8.4)	261 ± 144 CPZE	SZ = 12.3 (2.8); HC = 12.5 (2.7)	Alouette	rate, accuracy, speed	No significant differences were found between SZ and HC in neither of the three variables examined.	PANSS overall scores did not correlate with any reading subscores. The hallucination scores correlated significantly with reading efficiency and speed. Relationship between medication and reading NE.	Groups did not differ in achieved education levels. Groups were significantly different in WAIS scores. Relationship between cognition and reading NE.	French
Dondé et al. [18] *Meta-analysis*	**SZ**	SZ = 30 (21/11); **HC = 28 (24/6)**	SZ = 39.4 (11.2); HC = 37.2 (10.2)	NR	SZ = 14.1 (2.5); HC = 14.9 (2.0)	CTOPP (3 subscales), WJTA-III (3 subscales)	CTOPP PA, PM, APA: phonological processing; WJTA-III: comprehension, fluency, (BRS) – single-word reading	SZ had impaired phonological awareness for words and nonwords whereas phonological memory was intact. Reading comprehension and fluency were also significantly impaired. Single-word reading was intact in comparison to HC.	NE	MCCB correlations with reading skills were not reported. Groups did not differ in achieved education levels.	English
2. Affective disorders											
Weiss et al. [66]	**DD**	DD-intervention =38 (22/16); DD-control = 32 (17/15)	DD intervention = 41.4 (14.3); DD control = 43.7 (15.3)	NR	NR	REALM	single-word reading (literacy)	Only patients with limited literacy (scoring <60) were included. Literacy skills improved in DD intervention group after literacy training, and the depression severity lessened.	No correlation between depression symptoms (PHQ-9) and REALM at baseline. Relationship between medication and reading NE.	NE	English
3. Personality disorders/psychopathy											
Daderman et al. [15] *Forensic* *Meta-analysis*	**PD**	PD = 10 (7 dyslexia) (10/0); FC dyslexia = 26 (26/0); FC = 31 (31/0); **HC = 77** (77/0)	PD = 38.7 (5.89); FC = 35.1 (10.5); HC = 31.2 (10.8)	NR	PD = 9.8 (2.5); FC dyslexia = 9.1 (1.5); FC = 10.4 (2.1); HC = 11.1 (1.6)	“Summer with Monika”, MST, MWDT, JDT (Word chains)	“Summer with Monika”: reading speed, comprehension; MST: spelling; MWDT: reading pronunciation; JDT: word decoding	Dyslexia remains underdiagnosed in forensic psychiatric patients. 7/10 of forensic participants had dyslexia. Reading speed was slower in PD with dyslexia. Verbal comprehension was normal. PD with dyslexia scored significantly lower than FC without dyslexia and HC on measures of decoding and spelling and significantly poorer than HC in reading out loud. Reading was characterized by distortion and misreading.	NE	Patients had reading skills below their education levels. Relationship between cognition and reading NE.	Swedish
Brites et al. [30] *Forensic* *Meta-analysis*	**Psychopathy**	Psychopathy = 13; Psychopathy-Forensic =13; FC = 25 (51/0); **HC = 39**	38.19 (7.67)	NR	*M* = 9.3 (1.88)	PALPA	phonological processing, reading pronunciation and writing, comprehension of words and images, comprehension of sentences	Phonological processing and single-word reading were similar between psychopaths (forensic + nonforensic) and nonpsychopaths (forensic + nonforensic). Phonological processing was lower in imprisoned participants. Comprehension was also intact in psychopaths.	NE	Groups did not differ in achieved education levels. Relationship between cognition and reading NE.	Portuguese
Davidson et al. [68] *Forensic*	**ASPD**	ASPD: Research Naive = 18 (18/0); Research Experienced = 7 (7/0)	Research naive = 38.67 (9.7); Research experienced = 38.86 (8.0)	NR	NR	TOWRE	single-word reading (literacy)	Research experienced participants had higher literacy scores than research naïve ones. Participants with lower literacy prefer shorter wording and answered fewer questions correctly. Understanding of research terms may infer a higher ability to integrate research information.	NE	NE	English
4. General mental illness											
Berg and Hammitt [51]	**MI**	Alc = 53; PD = 6; Psychosis =30; Mental Retardation = 5; Organic Brain Syndrome = 6 (all: 74/26)	39	NR	MI = 9.0	PIAT (2 subtests)	comprehension, single-word reading (word recognition)	Over 50% of the patients scored below 7th grade in comprehension, resulting in being functionally illiterate. Patients scored significantly worse in comprehension than in single-word reading. Therefore, they could have read the text but did not understand it. Formal education was an indicator of word pronunciation but not comprehension. PD and Psychosis groups scored similarly in single-word reading and comprehension. Mental retardation and organic brain syndrome performed significantly lower than PD and Psychosis groups.	NE	Formal education was a good predictor of single-word reading but not for comprehension. Relationship between cognition and reading NE.	English
Dalby and Williams [70] *Forensic* *Meta-analysis*	**MI**	SZ = 30 (29/1); BD Manic = 15 (9/6); Alc = 28 (26/2); ASPD = 17 (17/0); **HC = 21** (21/0)	SZ = 29.37 (5.94); BP = 31.69 (9.37); Alc = 39.00 (11.54); ASPD = 25.53 (5.59); HC = 30.33 (10.31)	NR	SZ = 10.73 (2.60); BP = 11.07 (2.44); Alc = 9.54 (1.53); ASPD = 8.41 (2.12); HC = 10.43 (1.16)	WRAT	single-word reading (word recognition), spelling, arithmetic	Significant differences in reading, spelling, and arithmetic between all groups. Reading scores: Mania > SZ > HC > Alc > ASPD>	NE	In HC, IQ correlated with reading and spelling. Reading was significantly better than full-scale IQ in SZ and BD. Reading and spelling were preserved in psychotics despite lowered general IQ. Relationship between education and reading NE.	English
Nestor et al. [71] *Forensic*	**MI**	MI = 40: Young = 22 (22/0); Old = 18 (18/0)	MI Young = 19.3; MI Old = 41.4	NR	NR	WRAT-R	single-word reading, spelling, arithmetic	Violent patients: MI-Old scored significantly higher in WRAT-R reading subtest than MI-Young, suggesting developmental learning disability. Scores in Spelling and Arithmetic were not significantly different. Murder: MI-Old scored significantly higher in reading and spelling than MI-Young. Scores in arithmetic were not significant. Learning disability and conduct disorder may increase the probability of violence in MI-Young.	NE	NE	English
Christensen and Grace [65]	**MI**	SZ = 7; AfD = 27; AdjD = 2; Other = 9 (all: 32/13)	32	NR	NR	REALM	single-word reading (word recognition and pronunciation)	Over 75% of MI have reading skills on the level of 7th or 8th grade. People with MI are usually unaware of their reading problems. Reading screening recommended in routine evaluations.	NE	NE	English
Ferron et al. [58]	**MI**	SZ/SZAD = 95; Mood disorder = 34; Other MI = 6 (all: 97/38)	35 (10.0)	NR	NR	WRAT-IV	comprehension	WRAT reading and comprehension on the level of 9th grade of education.	NE	NE	English
Selenius et al. [26] *Forensic*	**MI with Psychopathy**	MI = 40: violence =29; sexual = 8; other = 3 (all: 32/8)	36 (10.0)	NR	MI = 10.04 (1.79)	MWDT, MST, The Hedgehog, MSVT (all tests by Madison), “The Pidgeon”, JDT	The Pidgeon: phonological processing; MWDT and JDT: word decoding; MST: spelling; The Hedgehog: reading speed, and comprehension; MSVT: vocabulary	Antisocial traits are not associated with reading. However, affective and interpersonal (Factor 1) traits were significantly related to decoding, reading speed and phonological processing. Phonology, semantics and syntactic skills significantly positively correlated with Superficial traits in psychopaths with MI.	Antisocial lifestyle did not correlate with reading skills. Affective and personality traits significantly positively correlated with sentence decoding and reading speed. Relationship between medication and reading NE.	NE	Swedish
Svensson et al. [27] *Forensic*	**MI**	MI = 185: Neurodevelopmental disorder = 58; DD = 40; Psychosis = 57; Anxiety = 13; PD = 12 (all: 133/52)	33 (9.9)	NR	NR	JDT (wordchains), word attack, phonological choice, orthographic choice, WRMT (oral close), RAN	JDT: decoding; word attack, phonological choice: phonological decoding; orthographic choice: spelling; oral close, RAN: reading comprehension	Low reading abilities interfere with psychiatric treatment in forensic mental health facilities. 16% of patients had a dyslexic profile. Psychosis and anxiety have the lowest general reading skills (phonological processing + comprehension). DD had a significantly better word, nonword reading, and comprehension than psychosis. General reading skills could not predict diagnoses.	NE	NE	

Abbreviations: AdjD, Adjustment Disorder; AfD, Affective Disorder; Alc, Alcoholism; BD, Bipolar Disorder; BDAE, Boston Diagnostic Aphasia Examination; BPRS, Brief Psychiatric Rating Scale; CPZE, Chlorpromazine equivalents; CTOPP, Comprehensive Test of Phonological Processing (PA, Phonological Awareness, PM, Phonological Memory, RN, Rapid Naming, APA, Alternative Phonological Awareness, ARN, Alternative Rapid Naming); DD, Depressive Disorder; FC, Forensic Controls (history of violence without MI); GORT, Gray Oral Reading Test; HC, Healthy Controls; ITBS, Iowa Test of Basic Skills; ITED, Iowa Test of Educational Development; JDT, Jacobson’s Decoding Test; LNNB, Luria-Nebraska Neuropsychological Battery; Mac-CAT-CR, MacArthur Treatment Competence Assessment Tool for Clinical Research; MCCB, MATRICS Consensus Cognitive Battery; MI, Mental Illness; MST, Madison’s Spelling Test; MSVT, Madison’s Standardized Vocabulary Test; MWDT, Madison’s Word Decoding Test; NARA, Neale Analysis of Reading Ability; NDRT, Nelson–Denny Reading Test; NE, Not Examined; NR, Not Reported; PALPA, Psycholinguistic Assessments of Language Processing in Aphasia; PANNS, Positive and Negative Syndrome Scale; PD, Personality Disorder; PIAT, Peabody Individual Achievement Test; RAN, Rapid Automatised Naming; RCBA, Reading Comprehension Battery for Aphasia; REALM, Rapid Estimate of Adult Literacy in Medicine; RNRT, Roentgen’s Nonwords Reading Test; RNST, Roeltgen’s Nonwords Spelling Test; SZ, Schizophrenia; SZAD, Schizo-Affective Disorder; TOWRE, Test of Word Reading Efficiency; WJCog, Woodcock–Johnson Test of Cognitive Ability; WJTA-III, Woodcock–Johnson III Tests of Achievement (BR, Broad Reading, BRS, Basic Reading Skills, RC, Reading Comprehension, PKG, Phoneme-Grapheme Knowledge); WRAT, Wide Range Achievement Test; WRMT-R, Woodcock Reading Mastery Test—Revised (BS, Basic Skills, PC, Passage Comprehension, PKG, Phoneme-Grapheme Knowledge).

Studies including forensic populations are marked *Forensic*.

Studies included in the meta-analysis are marked “*Meta-analysis*”.

Bold entries indicates Visual aid to distinguish studies using a control group as a reference

Studies that reported means and standard deviations (s.d.) for patient and healthy control (HC) groups to permit the calculation of effect sizes were included in the meta-analysis (effect sizes also presented where only one study available). The remaining studies contributed only to the narrative synthesis (see Table 3 for details). Studies assessing individuals with conditions primarily classified as neurodevelopmental (ADHD, autism, learning difficulties, and intellectual disabilities) [82] were excluded.

### Statistical analysis

The meta-analysis was conducted using Review Manager 5.3 Software—RevMan [83]. For eligible studies, effect sizes were calculated as Hedge’s g (standardized mean difference). A random-effects model was used as a more conservative approach. Heterogeneity was calculated as the *I^2^* measure of consistency for each meta-analytic calculation. Planned analyses included comparing each diagnosis (SZ, bipolar disorder, depression, anxiety, PDs, psychopathy), and unspecified general MI with healthy groups on specific reading skills (phonological processing and decoding; comprehension; single-word reading; rate, speed, accuracy, and fluency). For each reading skill, differences between tests to assess deficits in the patient group were calculated by investigating overlaps of confidence intervals of the summary effect sizes for each test. Risk of publication bias (none identified) was formally assessed via Egger’s and Begg’s tests and with funnel plots.

## Results

**Figure 1.**
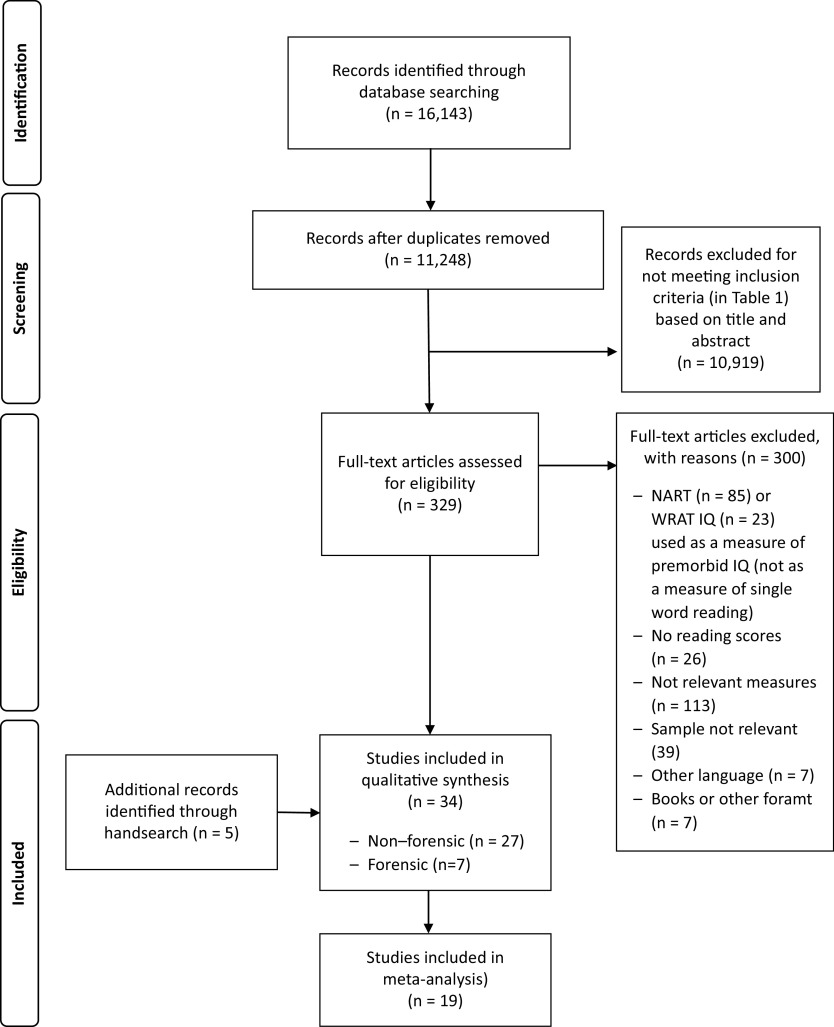
PRISMA flowchart.

Of 34 studies in total (Tables 2–3), 19 studies provided data for meta-analysis (Figure 1*. PRISMA flowchart*); five of these studies also presented composite scores (combining two or more measures) that are covered in the narrative synthesis. The remaining 15 studies contributed to the narrative synthesis only. The findings from the nonforensic and forensic samples are presented separately, followed by a direct comparison of forensic and nonforensic groups.

## Reading Skills in Nonforensic Populations

### Schizophrenia

*Phonological Processing and Decoding:* Across seven studies (Figure 2(2.1)), SZ showed significantly poorer phonological processing compared to HC with a large effect size (Hedge’s *g* = −0.88, *df* = 24, *p* < 0.00001, CI = [−1.07, −0.70]). There was medium heterogeneity within the data (*p* = 0.001, *I*^2^ = 53%), with nonsignificant differences between the tests (*p* = 0.15, *I*^2^ = 32.3%).Figure 2.Reading deficits in schizophrenia (non-forensic population). Within each specific reading skill, the results are presented for each of the test(s)/measures used, followed by the analysis of differences between tests (last row). Negative values represent a poorer performance of people with schizophrenia in comparison to HC.References: Arnott et al. [24]; Curzietti et al. [76]; Dondé et al. [18]; Gavilán and García-Albea [39]; Hayes and O’Grady [54]; Ho et al. [45]; Leonard et al. [36]; Light et al. [74]; Maj [62]; Martinez et al. [41]; Patrick et al. [59]; Potter and Nestor [73]; Revheim et al. [21]; Revheim et al. [17]; Walder et al. [22]; Whitford et al. [7]. Abbreviations: BDAE, Boston Diagnostic Aphasia Examination; CTOPP, Comprehensive Test of Phonological Processing (PA, Phonological Awareness, PM, Phonological Memory, RN, Rapid Naming, APA, Alternative Phonological Awareness, ARN, Alternative Rapid Naming); GORT, Gray Oral Reading Test; ITBS, Iowa Test of Basic Skills; ITED, Iowa Test of Educational Development; LNNB, Luria-Nebraska Neuropsychological Battery; NARA, Neale Analysis of Reading Ability; NDRT, Nelson–Denny Reading Test; PALPA, Psycholinguistic Assessments of Language Processing in Aphasia; RAN, Rapid Automatised Naming; RCBA, Reading Comprehension Battery for Aphasia; RNRT, Roentgen’s Nonwords Reading Test; RNST, Roeltgen’s Nonwords Spelling Test; WJTA-III, Woodcock-Johnson III Tests of Achievement Knowledge); WRAT, Wide Range Achievement Test; WRMT-R, Woodcock Reading Mastery Test-Revised. White circle 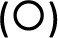
—effect size for a particular study determining the difference between patients and controls. Black diamond 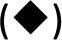
—pooled effect size for particular test/subtest. Red diamond 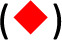
—overall effect size for diagnosis for a certain reading skill (e.g., comprehension) including all partial effect sizes.

**2.1.**
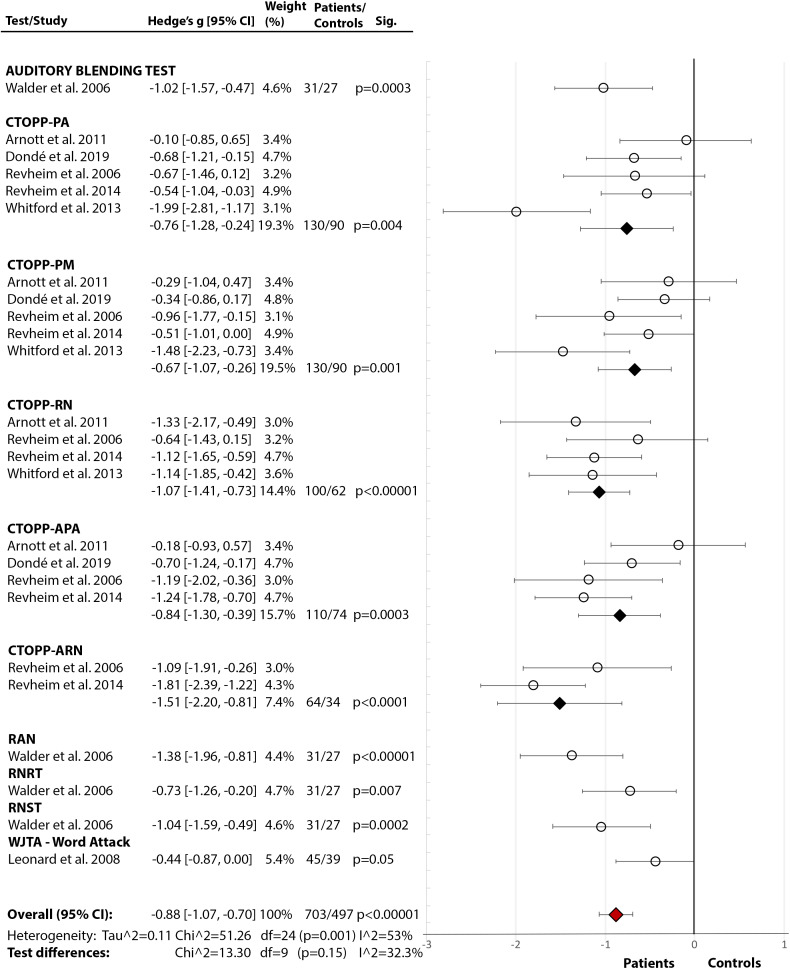
Phonological processing and decoding

**2.2.**
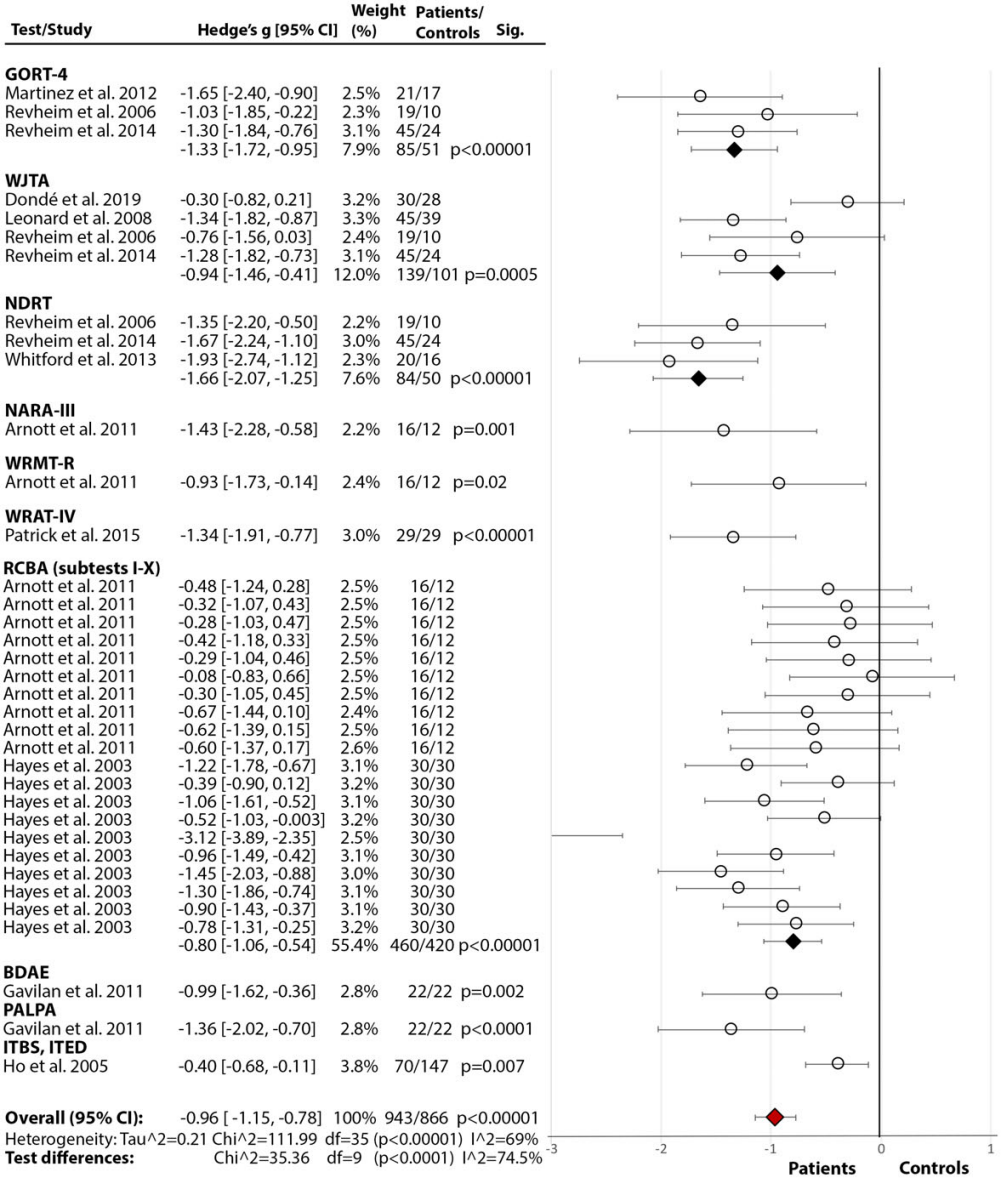
Comprehension

**2.3:**
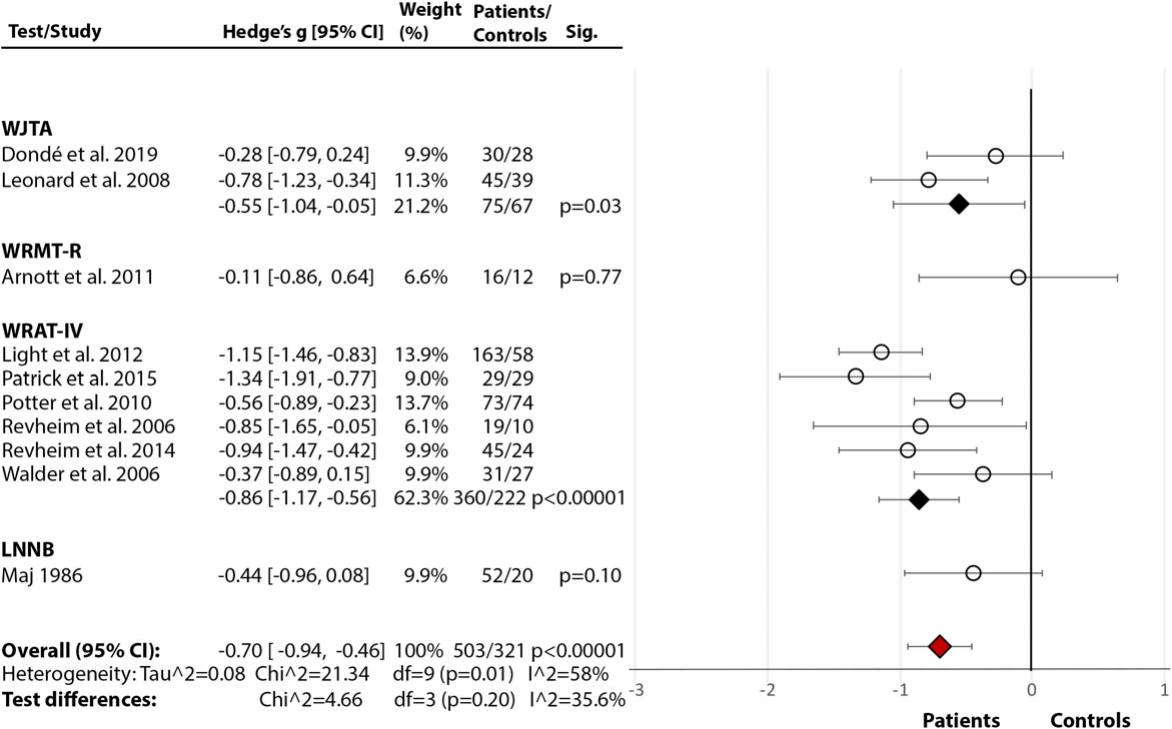
Single-word reading

**2.4.**
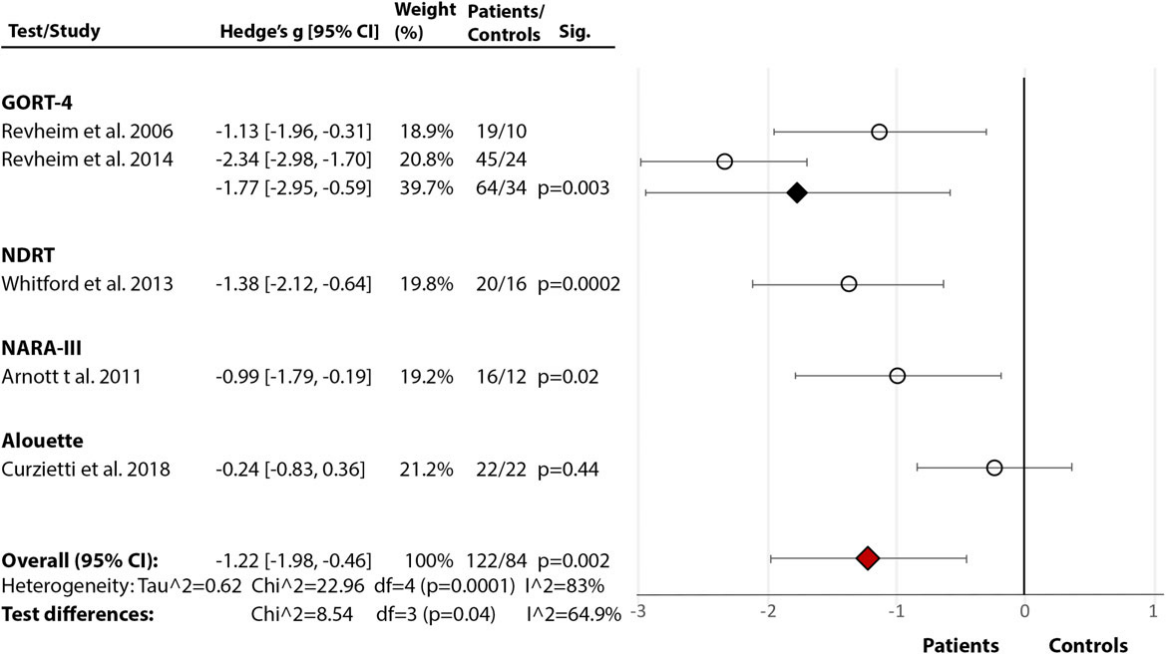
Rate

**2.5:**
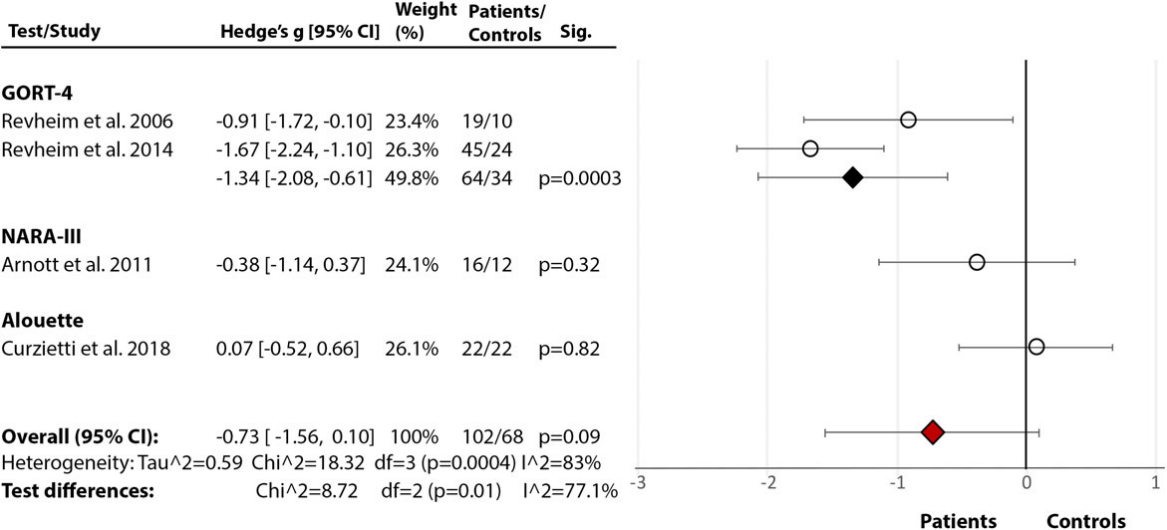
Accuracy

**2.6:**
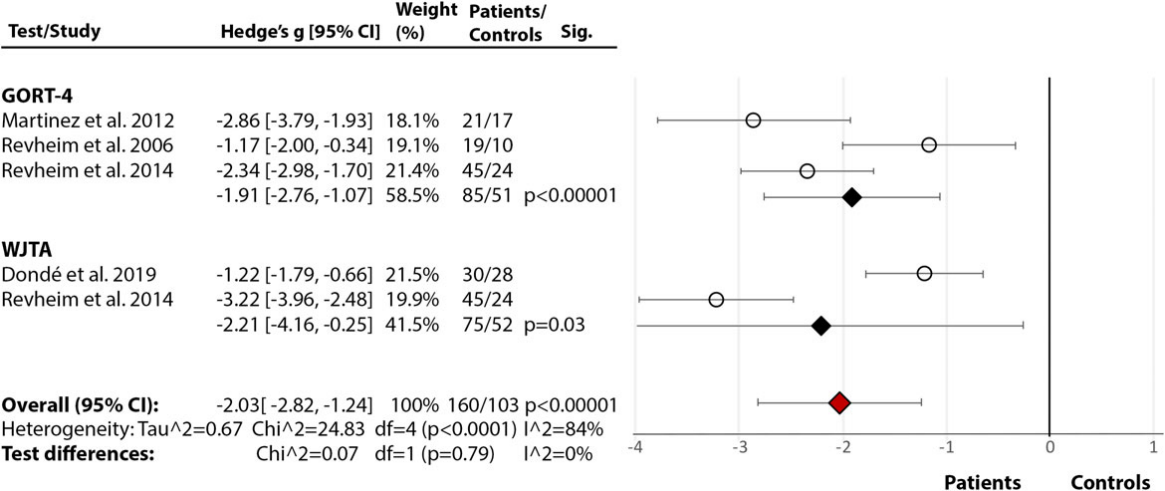
Fluency

**2.7:**
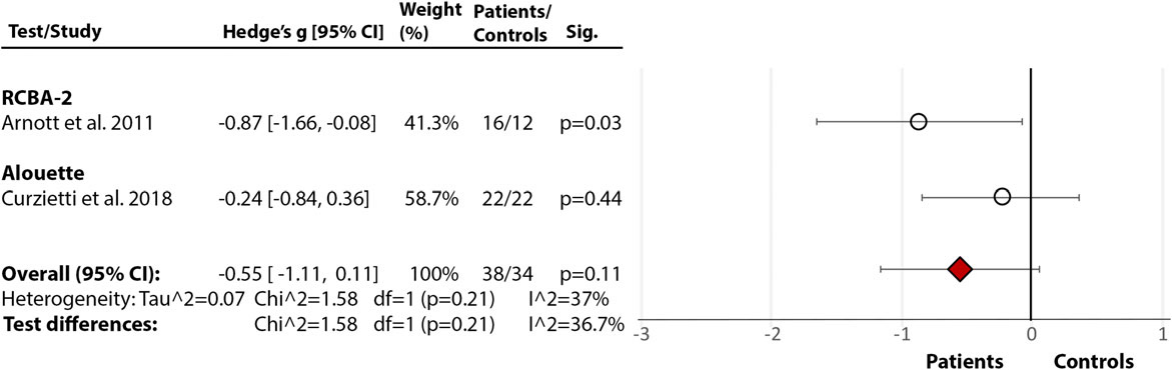
Speed

*Comprehension:* Across 11 studies (Figure 2(2.2)), SZ showed poorer comprehension than HC with a large overall effect size (Hedge’s *g* = −0.96, *df* = 34, *p* < 0.00001, CI = [−1.15, −0.78]) and medium heterogeneity (*p* < 0.00001, *I*^2^ = 69%). The test differences were significant (*p* < 0.0001, *I*^2^ = 74.5%) with NDRT [49] and GORT-4 [40] showing the largest effect sizes for a comprehension deficit in SZ. In addition, three studies [17,21,41] reported lower Oral Reading Quotient from GORT-4 [40]. In other studies, retrospective assessment revealed that those with a current diagnosis were below the norm during 4th to 11th grade of school [44], with the most prominent deficit in the 11th grade, indicating a gradual decline [44,45]. A similar study on adolescents, who later developed psychosis, displayed a premorbid deficit in comprehension and sentence reading relative to HC [47].

*Single-Word Reading:* Across 10 studies [17,18,21,22,24, 36,59,62,73,74], there was a significant medium-size deficit (Figure 2(2.3)) in SZ relative to HC (Hedge’s *g* = −0.70, *df* = 9, *p* < 0.00001, CI = [−0.94, −0.46]). There was significant heterogeneity within the results (*p* = 0.01, *I*^2^ = 58%) but no test performed better than others (*p* = 0.20, *I*^2^ = 35.6%). Moreover, in two studies [62,63], both using LNNB—Reading subtest (see Table 2 for test descriptions) [61]—SZ showed a deficit compared to HC (data for meta-analysis not provided). In a third study [72], SZ scored markedly lower (*M* = 78.00, SD = 21.01) than the norm (*M* = 100) on WRAT-III [84].

*Rate, Speed, Accuracy, and Fluency:* Across five studies [17,21,24,76,85], there was a significant large effect of SZ diagnosis on reading rate (Hedge’s *g* = −1.22, *df* = 4, *p* = 0.002, CI = [−1.98, −0.46]) (Figure 2(2.4)). The effect of diagnosis [17,21,24,76] in accuracy failed to reach significance (Hedge’s *g* = −0.73, *df* = 3, p = 0.09, CI = [−1.56, 0.10]) (Figure 2(2.5)). There were, however, significant test differences for both rate (*p* = 0.04, *I*^2^ = 64.9%) and accuracy (*p* = 0.01, *I*^2^ = 77.1%), with the GORT-4 revealing large deficits [17,21], and the Alouette [75] showing no deficit [76] (Figures 2(2.4–2.5)). In fluency [17,18,21,41,77], there was a highly significant deficit in SZ (Hedge’s *g* = −2.03, *df* = 4, *p* < 0.00001, CI = [−2.82, −1.24]), but with large heterogeneity within results (84%) (Figure 2(2.6)). In reading speed (time taken to read certain content) [24,76], the effect of diagnosis was nonsignificant (Hedge’s *g* = −0.50, *df* = 1, *p* = 0.11, CI = [−1.11, −0.11]) (Figure 2(2.7)). In an additional study [77], 10–11% of SZ demonstrated nonfluencies (e.g., sound repetitions at beginning of word) in sentence and paragraph reading during the BDAE [38].

*Composite Scores:* Two studies [17,21] that examined Basic Reading Skills (phonological processing and single-word reading) and Phoneme-Grapheme Knowledge (phonological processing and orthography) composite scores from WJTA-III [35] showed different results, with only one of these showing a significant deficit in SZ [17]. Both studies [17,21] found significantly lower WJTA-III Broad Reading (phonological processing, comprehension, speed) scores in SZ, relative to HC. The study [22] that created a phonology composite score by combining the RNRT [33], RNST [33], WRAT-R [86], and the Controlled Oral Word Association Test (COWAT) [87] also reported a significant deficit in SZ relative to HC.

#### Reading-related skills

*Vocabulary*: Six studies [17,21,22,44,45,81] assessed reading-related skills in SZ. There was evidence of impaired vocabulary from an early age [44,45] and those with prodromal illness scored significantly below grade-norms when assessed by the ITBS [42] and ITED [43] as a part of their school performance. Vocabulary, assessed using the NDRT [49], was also impaired in two studies [17,21].

*Spelling and Grammar*: Spelling in RNST [33] was found to be adversely affected in male patients, while female patients scored similarly to HC [22]. Another study [44], which longitudinally assessed spelling together with grammar and other language-related skills by ITBS [42], found a significant decline in abilities at 11th grade in SZ. Similarly, SZ scored significantly lower in the WJTA-III [35] spelling subtest compared to HC [17]. Grammar was assessed exclusively in one study [22], using Caplan and Hildebrandt’s task [79], showing a stronger and significant deficit in male, relative to female, patients [22].

*Orthography*: Orthography processes are not reading abilities. However, in languages such as Chinese, orthography and semantics play an important role in reading, in contrast to alphabetical languages such as English where phonological processing plays a key role [81]. One study [81] that investigated orthography processes found significant deficits in orthography-phonology, but not in vocabulary when distinguishing real words from nonwords, in SZ compared to HC.

### Affective disorders (depression, anxiety or mania)

Two studies [62,66] assessed single-word reading in depression, both using the REALM [64]. Of these, one study [62] showed a nonsignificant small deficit in people with depression (Hedge’s *g* = −0.30, *df* = 0, *p* = 0.37, CI = [−0.96, 0.36]) and, in the other study [66], all participants performed at 7–8th grade reading level.

### Bipolar disorder

The earlier-mentioned study on adolescents [47] had also assessed comprehension premorbidly in a group who later developed nonpsychotic bipolar disorder and found them to have no deficit in comparison to HC.

### Personality disorders/psychopathy

One study [30] assessed phonological processing and comprehension, using the Portuguese version of the PALPA [29], and showed medium-size deficits in both phonological processing (Hedge’s *g* = −0.55, *df* = 2, *p* = 0.004, CI = [−0.92, −0.18]) (Figure 3(3.1)) and comprehension (Hedge’s *g* = −0.47, *df* = 0, *p* = 0.05, CI = [−0.87, 0.39]) (Figure 3(3.2)) in people with diagnosed psychopathy (from community settings), compared with nonpsychopathic nonforensic controls.Figure 3.Reading deficits in community/nonforensic samples of people with psychopathy. Within each specific reading skill, the results are presented for each of the test(s)/measures used, followed by the analysis of differences between tests (last row). Negative values represent a poorer performance of people with personality disorder in comparison to healthy control.Brites et al. [30]. Abbreviations: PALPA, Psycholinguistic Assessments of Language Processing in Aphasia. White circle 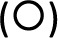
—effect size for a particular study determining the difference between patients and controls. Red diamond 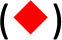
—overall effect size for diagnosis for a certain reading skill (e.g., comprehension) including all partial effect sizes.

**3.1:**
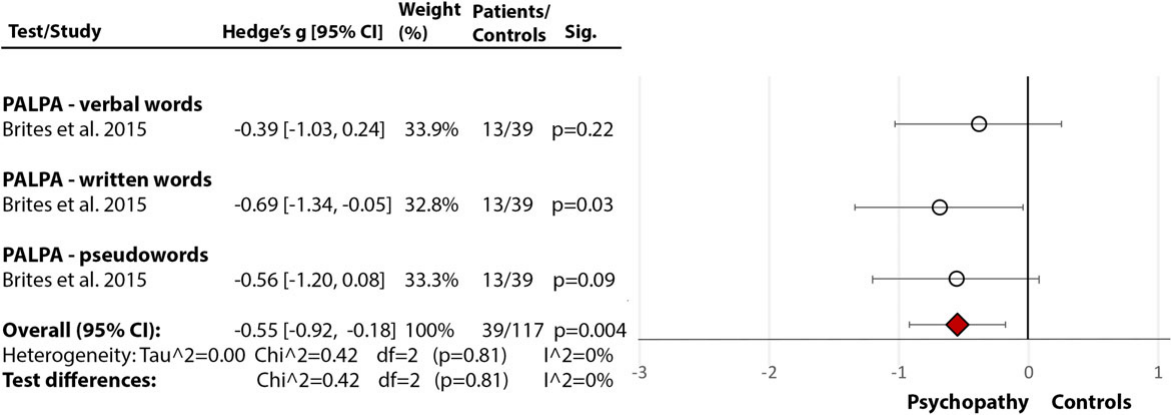
Phonological processing and decoding.

**3.2:**
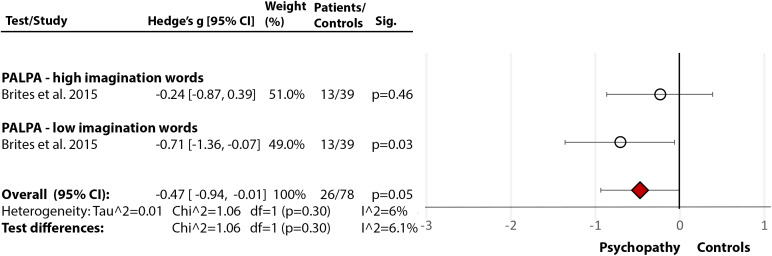
Comprehension.

### General mental illnesses (nonspecified/mixed)

Two studies [51,58] assessed comprehension and single-word reading while the third study [65] assessed single-word reading only. The first study [58] reported 9th-grade level comprehension as well as 9th-grade level single-word reading when assessed by WRAT-IV [88] in people with unspecific MIs. The second study [51], using the PIAT-comprehension subtest [50], reported 7th-grade comprehension, despite 9–10th grade for single-word reading, in psychiatric patients (majority with alcoholism or nonorganic psychoses). In the third study [65], 75% of the sample with MIs (mainly SZ and affective disorders) read below 7th grade when assessed by REALM [64].

## Summary of Deficits in Nonforensic Populations

**Figure 4.**
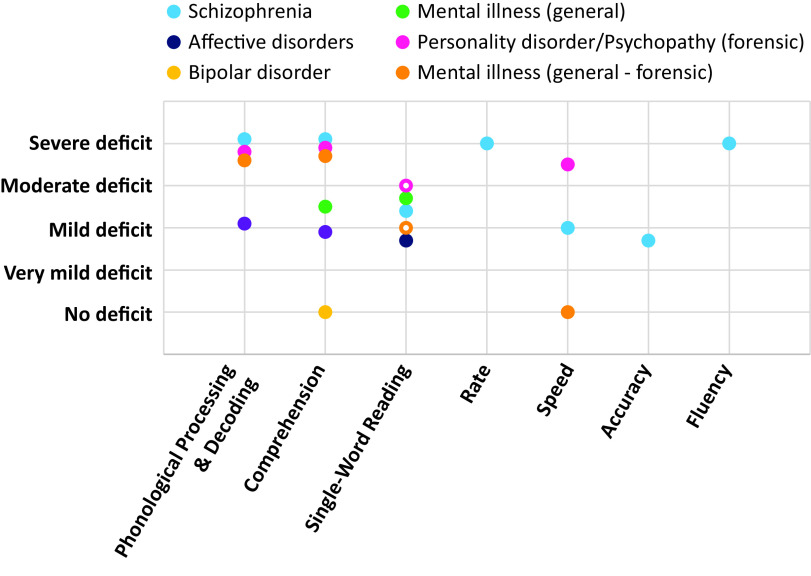
Interpretation of observed reading deficits in included diagnoses. No deficit = nonsignificant differences between patients and healthy control (HC); Very mild deficit = Hedge’s g up to −0.30 and/or mixed results with the majority of samples scoring within the norm; Mild deficit = Hedge’s g up to −0.50 and/or reading skill at 9–10th-grade level; Moderate deficit = Hedge’s g up to −0.75 and/or reading skill at 7–8th grade level; Severe deficit = Hedge’s g over −0.75 and/or reading skill below 7th grade level. This interpretation considers whether the results were consistent or mixed. Empty circle 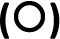 = Mixed evidence.

Overall, SZ was associated with pronounced deficits in phonological processing, comprehension, reading rate, and fluency (Figure 4), with deficits also present in reading-related skills. These deficits appear to be present often from an early age, with reading skills of SZ adults remaining below their achieved education levels. The single-word reading and speed were less impacted. There were few data in affective disorders, and only for single-word reading, showing a mild/nonsignificant deficit. Individuals with PDs/high psychopathy showed mild deficits in both phonological processing and comprehension (Figure 4). Comprehension and single-word reading skills of people with unspecified MIs from nonforensic settings were at secondary school levels, which, although below the norm, were better than those in SZ (Figure 4).

## Reading Skills in Forensic Populations

Seven studies [15,26,27,30,68,70,71], all in PDs/psychopathy or general MIs, were found.

### Personality disorders/psychopathy

*Phonological Processing and Decoding:* In the first study [30], the PALPA [29] phonological processing test showed a large deficit in the incarcerated group with diagnosed psychopathy relative to HC (Hedge’s *g* = −0.85, *df* = 2, *p* = 0.0001, CI = [−1.22, −0.47]) (Figure 5(5.1)). The second study [15], using the JDT [25] to examine decoding, showed marked impairment (Hedge’s *g* = −0.84, *df* = 0, *p* = 0.01, CI = [−1.51, −0.17]) in people with nonspecific PDs (and comorbid MIs), relative to HC.Figure 5.Reading deficits in forensic patients with psychopathy or personality disorders. Within each specific reading skill, the results are presented for each of the test(s)/measures used, followed by the analysis of differences between tests (last row). Negative values represent a poorer performance of people with psychopathy or personality disorder in comparison to healthy control.Brites et al. [30]; Daderman et al. [15]. Abbreviations: JDT, Jacobson’s Decoding Test; PALPA, Psycholinguistic Assessments of Language Processing in Aphasia. White circle 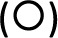
—effect size for a particular study determining the difference between patients and controls. Red diamond 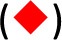
—overall effect size for diagnosis for a certain reading skill (e.g., comprehension) including all partial effect sizes.

**5.1.**
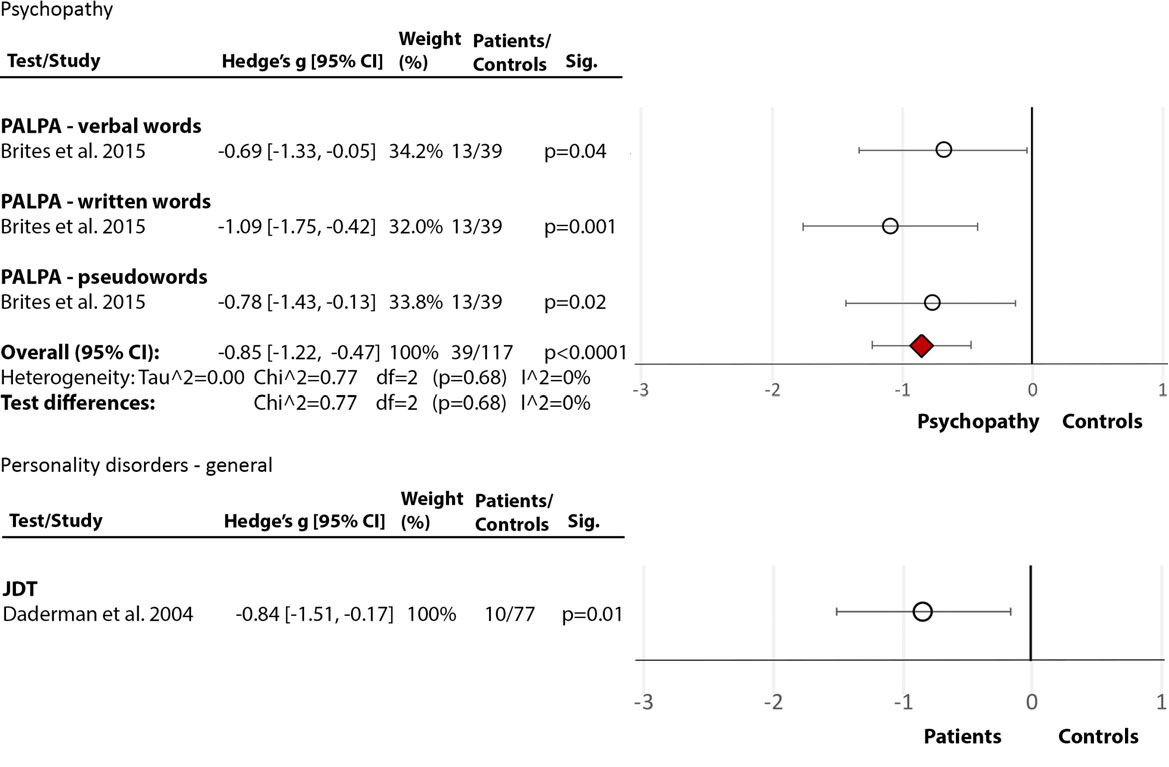
Phonological processing and decoding

**5.2.**
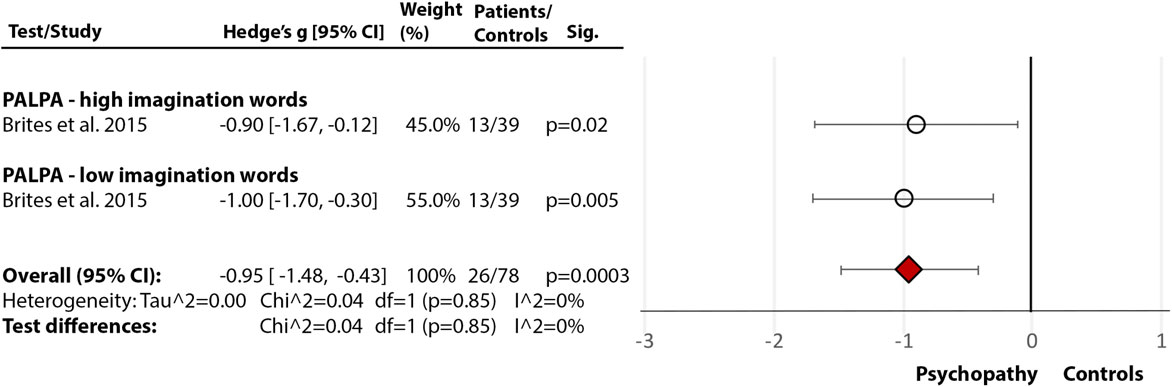
Comprehension. Psychopathy.

*Comprehension:* One study [30] used the PALPA [29] and showed a large deficit in comprehension in incarcerated people with diagnosed psychopathy, compared to HC (Hedge’s *g* = −0.95, *df* = 0, *p* = 0.0003, CI = [−1.48, −0.43]) (Figure 5(5.2)). The other study [15] used a Swedish prose text [55] and found no deficit in PDs.

*Single-word Reading:* The first study [15] used a Swedish single-word reading test [28] and found significant impairment in PD inmates with comorbid MI and dyslexia, as well as in dyslexic inmates, in comparison to inmates without a PD diagnosis. In the second study [30], a diagnosis of psychopathy did not influence single-word reading as assessed by PALPA [29]. The third study [68] found literacy scores, as assessed by the TOWRE [67], to be below the norm in PD. None of these studies [15,30,68] provided data for effect size calculation.

*Rate, Speed, Accuracy, and Fluency:* Only one study [15] was found, showing that reading speed was negatively affected in 7 of 10 forensic PD participants, especially in those with comorbid dyslexia.

#### Reading-related skills

One study [15] showed that spelling was poorer in inmates with PD and dyslexia, as opposed to those with no comorbidities.

### General mental illnesses (nonspecified/mixed)

*Phonological Processing and Decoding:* One study [27] used the JDT–Wordchains [25], the Word Attack test [89], and Phonological Choice [31], and revealed severely impaired phonological skills (below the 6th grade) in people with various MIs. The second study [26] examined correlations between psychopathic traits and phonological and decoding skills in forensic psychiatric patients, assessed with the “Pidgeon” test [34], the MWDT [28], and the JDT [25], and found positive correlations between the superficial item of the Psychopathy Checklist: Screening Version (PCL:SV) [90] and phonological processing and decoding of sentences (but not words). However, as the study did not include HCs or test normative scores, the findings are difficult to understand in terms of quantifying the deficit.

*Comprehension:* In one study [27] that used the Oral Close subtest of the WRMT-R [37], comprehension in inmates with MI was below 4th grade in 23% of Swedish native and in over 50% of non-native speakers. In another study [26] that used a silent paragraph reading test [56], no significant correlations between psychopathic traits and comprehension scores in people with nonspecified MIs were found.

*Single-word Reading:* There were two studies [70,71], both using the WRAT [69]. The first study [70] assessed people with various diagnoses (psychosis, mania, alcoholism, and ASPD). It found no significant differences between HC and psychosis (Hedge’s *g* = 1.42, *df* = 0, *p* = 0.68, CI = [−5.40, 8.24]), mania (Hedge’s *g* = 0.53, *df* = 0, *p* = 0.13, CI = [−0.15, 1.20]), or alcohol abuse (Hedge’s *g* = −0.49, *df* = 0, *p* = 0.10, CI = [−1.06, 0.09]) but single-word reading was significantly impaired in ASPD (Hedge’s *g* = −1.01, *df* = 0, *p* = 0.004, CI = [−1.69, −0.33]. The second study [71] found age-moderated differences in people with MIs and a history of violence, with people aged above 45 years scoring significantly better than those below 20 years.

*Rate, Speed, Accuracy, and Fluency:* One earlier-described study [26] found that, within those with MIs, reading speed [56] was positively correlated with affective and interpersonal traits (Factor 1, PCL:SV [90]).

#### Reading-related skills

In a study [26] involving Swedish inmates with MIs, neither spelling nor vocabulary scores significantly correlated with psychopathic traits.

## Summary of Deficits in Forensic Populations

Overall, there was evidence of severe impairment in phonological processing and decoding in forensic populations with PDs/psychopathy (Figures 4 and 5), similar to that seen in SZ. There was also evidence of deficits in comprehension, single-word reading, and speed in this population (Figures 4 and 5). Studies on forensic patients with various MIs yielded mixed findings although one study [27] that examined inmates did show phonological processing and comprehension to be well below the norm.

## Nonforensic versus Forensic Populations: Direct Comparison

Only one study [30] directly compared forensic and nonforensic groups. It used PALPA [29] and revealed a significant medium-size deficit in incarcerated individuals with psychopathy compared to nonincarcerated (community) sample with psychopathy in phonological processing and decoding (Hedge’s *g* = −0.49, *df* = 2, *p* = 0.03, CI = [−0.94, −0.04] (Figure 6(6.1)), and a large deficit in comprehension (Hedge’s *g* = −0.85, *df* = 1, *p* = 0.003, CI = [−1.43, −0.28]) (Figure 6(6.2)). These results support the findings from individual studies indicating severe reading deficits in incarcerated individuals with MI.Figure 6.Reading deficits in incarcerated vs community samples of people with a diagnosis of psychopathy. Within each specific reading skill, the results are presented for each of the test(s)/measures used, followed by the analysis of differences between tests (last row). Negative values represent a poorer performance of the forensic sample, compared to the nonforensic sample.Brites et al. [30]. Abbreviations: PALPA, Psycholinguistic Assessments of Language Processing in Aphasia. White circle 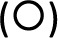
—effect size for a particular study determining the difference between patients and controls. Red diamond 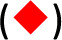
—overall effect size for diagnosis for a certain reading skill (e.g., comprehension) including all partial effect sizes.

**6.1:**
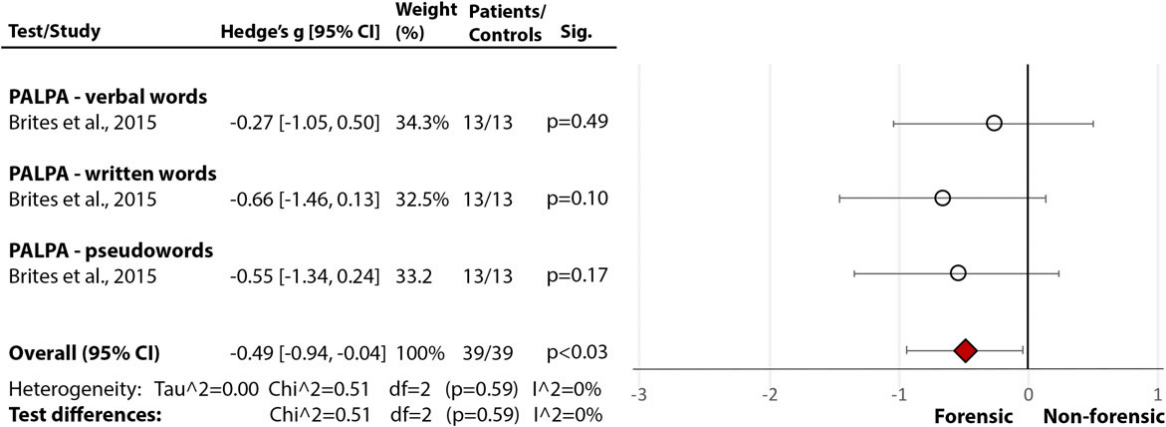
Phonological processing and decoding.

**6.2:**
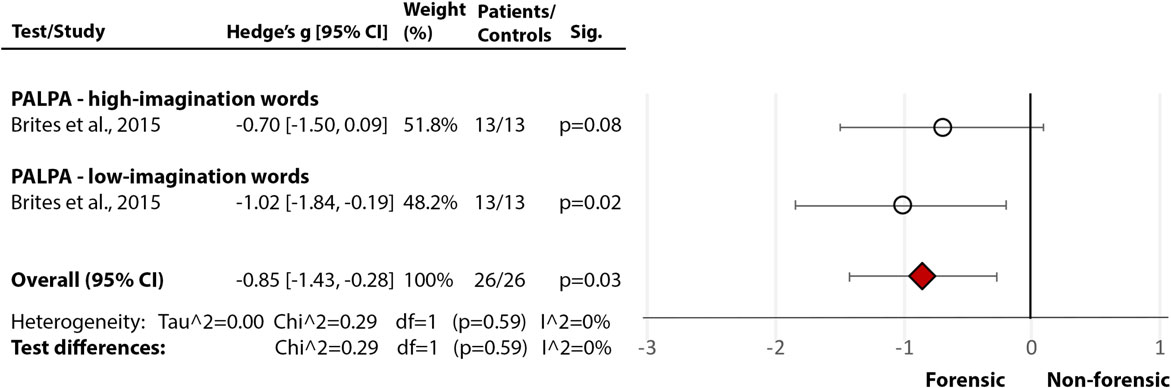
Comprehension.

## Reading Skills Deficits in Mental Illness: Influencing Factors

### Symptoms and medication

Of six studies in SZ [17,21,24,72,76,81] that examined the relationship between psychotic symptoms and reading skills, three [21,24,81] found a negative influence of positive and negative symptoms on phonological processing, comprehension, and orthography; and hallucinations negatively affected reading efficiency and speed in one study [76]. Five studies [17,22,41,63,85] examined the effect of antipsychotic dose as chlorpromazine equivalents; four [17,22,63,85] found no relationship with single-word reading, phonological processing, or comprehension, and one [41] found a negative influence of high dosage on fluency and comprehension. No significant association occurred between depressive symptoms and single-word reading [66].

### Cognitive function

Six studies [17,21,41,44,54,70] examined the relationship between reading skills and general cognition in SZ. Verbal IQ significantly correlated with comprehension and vocabulary [44]. Lower premorbid IQ (single-word reading) predicted reading comprehension [17,54]. However, general IQ did not significantly predict any of the reading skills [41]. Similarly, working memory did not correlate with comprehension or reading rate in SZ and HC [21]. In forensic populations, full-scale IQ was significantly lower than single-word reading in individuals with SZ and bipolar disorder [70]. These results suggest that general verbal skills may influence comprehension but no marked impact of other cognitive abilities was found.

### Education

In SZ, three studies [17,21,41] examined the influence of education and all found reading skills significantly below achieved academic levels. Six studies [18,24,39,62,76,81] matched their groups on education or entered it as a covariate [85], and all found significant impairments in various reading skills. Nonforensic populations with general MIs had single-word reading equivalent to their achieved education but their comprehension was lower [51]. Forensic PD also had comprehension below their education level [15].

## Discussion

This systematic review and meta-analysis evaluated existing evidence to identify the type and degree of reading impairments in different MIs, the reading assessment tools that might most consistently detect them, and possible differences in the pattern of reading skills deficits in people with different MIs in forensic and nonforensic settings. Most of the reviewed studies (27/34) included people with SZ. There were seven studies of reading skills deficits in people with different MIs (PD or general MI) in forensic settings. Our findings are discussed below.

### Effect of diagnosis in nonforensic samples

We observed significant deficits in multiple reading skills in SZ, resembling the pattern typically seen in dyslexia [6], and consistent with previous evidence for shared genetic and psychophysiological traits in SZ and dyslexia [7]. In our meta-analysis, both phonological processing and comprehension were greatly impaired. These impairments may be associated with ineffective use of contextual information [91] and contribute to poor speech in SZ, especially in close association with thought disorder [92]. Reading rate was low but the deficit in reading accuracy was lower. This indicates relatively preserved single-word reading skills, most likely because they are usually acquired before illness onset and remain intact [47]. In contrast, there was evidence for impairments in vocabulary and spelling, presumably as a result of disrupted scholastic experience. Disrupted scholastic experience during adolescence can affect complex skills such as comprehension [44,45,47], which could precipitate difficulties with processing complex written information in SZ. People with SZ showed reading skills well below their achieved education level (see *Education*). Reading skills deficits in SZ also do not seem to be explained by other aspects of cognition (see *Cognitive Function*) although more comprehensive investigations are needed to substantiate this. Our findings (*Symptoms and Medication*) further indicated that while symptoms and high antipsychotic doses may worsen reading skills, they do not fully explain the profile of reading skills deficits in SZ. Impairment in comprehension and vocabulary was present even before the onset of symptoms [44,45] together with deficient phonological processing, which has been related to disrupted visual processing in SZ since early age [21]. The symptoms can, however, aggravate deficits in reading skills, such as comprehension, which are acquired with experience, and also depend on the earlier acquired skills [93]. Recent data [94] suggest that some aspects of language production (e.g., slower articulation) that can affect reading skills assessments are particularly sensitive to dopamine-D2 receptor blocking antipsychotics. Furthermore, most studies in SZ included more men than women or men solely and also included people with schizoaffective disorder. Further studies need to comprehensively examine specific reading skills in both men and women with schizophrenia and schizoaffective disorder (separately) while taking medication, symptoms, cognition, education, and socioeconomic status into account.

Unlike in SZ and psychosis [51,58,65], nonpsychotic bipolar disorder, and affective disorders, seemed to have comprehension and single-word reading skills comparable to HC [30,47]. Although not all studies specified the type of PD, it seems that reading skill deficits may not be as prominent in nonforensic psychopathy as in SZ.

### Effect of diagnosis in forensic samples

Our findings suggest only a weak or no deficit in nonforensic psychopathy but indicate a marked phonological processing and comprehension deficit in the incarcerated group. It is possible that PD/psychopathic individuals with good phonological processing and comprehension are more able to evade incarceration [30,95]. Nonetheless, marked reading deficits in the incarcerated group may have contributed to their poor adjustment within the community [27], which, in turn, increased the risk of incarceration. Men with MIs within forensic settings had significantly lower general reading abilities and spelling than women with MIs [27], consistent with the pattern seen in healthy samples [22].

### Clinical implications

Comprehension has a significant influence on decision-making capacity in SZ [96], and this is likely to be true also for people with other MIs, especially within forensic populations. Dyslexia is often underdiagnosed in people with MIs, and this might explain their inability to complete higher education and obtain jobs [15], or the expression of socially unacceptable behaviors [27]. Furthermore, progression and engagement in therapeutic activities within mental health services often depend on good reading and language skills. This highlights a need to accurately identify reading deficits and develop specific programs to improve reading skills of people in psychiatric services. It may be possible to target reading deficits in SZ and other MIs by building on the less affected aspects, such as lexical knowledge (access to words) [97,98], and access to familiar information that can compensate for some of the reading deficits [99], while implementing interventions to ameliorate reading skills [100].

### Effect of assessments

Significant between-test differences were found only in tests detecting deficits in comprehension, accuracy, and rate in SZ. In comprehension and rate, the NDRT and GORT-4, and in accuracy, the GORT solely, consistently detected large deficits while the Alouette (French) test detected no deficits (Figure 2). It is conceivable that certain deficits emerge more often/strongly in English compared to some other languages, as is the case in developmental dyslexia [101]. This possibility requires further study.

## Conclusions

Our findings demonstrate pronounced deficits in phonological processing and comprehension in SZ and forensic PD/psychopathy. Reading skills in people with other MIs in nonforensic settings seem relatively unaffected. Among the tests, only the NDRT and GORT detected significantly stronger deficits in SZ than other measures. Considering the importance of good reading skills in everyday life, as well as for the clinical success of mental health services, there is a clear need to identify methods that can improve reading in SZ and forensic PD populations. These interventions could potentially build on relatively spared aspects of reading by implementing approaches already effective in dyslexia.

## Data Availability

All data supporting the meta-analysis reported in this article are available from Brunel University London research repository at 10.17633/rd.brunel.13123334.
